# Supplementary material for: Mechanotransduction is required for establishing and maintaining mature inner hair cells and regulating efferent innervation
Source: Nat Commun. 2018 Oct 1;9:4015. doi: 10.1038/s41467-018-06307-w (PMC6167318; doi:10.1038/s41467-018-06307-w)
Supplement: Supplementary file 1 — Supplementary Information [file 41467_2018_6307_MOESM1_ESM.pdf]

Mechanotransduction is required for establishing and maintaining mature inner hair cells and regulating efferent innervation

Corns et al.

Supplementary Table 1. Basolateral membrane properties from immature IHCs of *Ush1c*$^{fl/fl}$ conditional knockout mice

Pre-hearing IHCs

	***Ush1c*$^{fl/fl}$ (P7)**		***Ush1c*$^{fl/fl}$ (P9)**		***Ush1c*$^{fl/fl}$ (P10)**	
	Ctrl	Cre+	Ctrl	Cre+	Ctrl	Cre+
Resting potential (mV)	-63.2 ± 2.4 (3)	-61.2 ± 1.4 (6)	-70.0 ± 4.0 (3)	-76.1 ± 0.8 (4)	-77.1 ± 1.3 (5)	-78.4 ± 0.7 (7)
Membrane capacitance (pF)	7.7 ± 0.1 (3)	7.6 ± 0.2 (6)	8.6 ± 0.6 (3)	9.3 ± 0.1 (4)	10.0 ± 0.4 (5)	10.5 ± 0.5 (7)
I_{K1} at -124 mV (pA)	-430 ± 46 (3)	-475 ± 33 (6)	-362 ± 97 (3)	-234 ± 18 (4)	-495 ± 29 (5)	-423 ± 39 (7)
I_K at 0 mV (nA)	4.0 ± 0.4 (3)	4.3 ± 0.4 (6)	5.2 ± 0.5 (3)	5.4 ± 0.4 (4)	7.7 ± 0.4 (5)	7.3 ± 0.5 (7)
Adult type K$^+$ currents						
$I_{K,f}$ at -25 mV (nA)	-	-	-	-	-	-
$I_{K,n}$ at -124 mV (pA)	-	-	-	-	-	-

Values are means ± s.e.m.; number of hair cells are in parentheses. I_{K1} = Inward rectifier K$^+$ current[1]; I_K = Delayed rectifier K$^+$ current[2]; $I_{K,n}$ = Negatively activated K$^+$ current carried by KCNQ4 channels[2]; $I_{K,f}$ = Ca^{2+}-activated K$^+$ current[3]. "-" = not present. The size of the outward I_K (steady-state at 0 mV) and the inward I_{K1} (steady-state at –124 mV) was obtained from holding potentials of –84 mV and –64 mV, respectively. No significantly different values were found between control (Ctrl, *Ush1c*$^{fl/fl}$: 3 mice) and knockout (Cre$^+$, *Ush1c*$^{fl/fl}$*Myo15*-cre$^{+/-}$: 6 mice) IHCs at each age tested (P7, P9, P10) using Student's *t* test.

Supplementary Table 2. Basolateral membrane properties from mature IHCs of *Ush1c*$^{fl/fl}$ conditional knockout mice

Adult

	***Ush1c*$^{fl/fl}$ (P14-P15)**		***Ush1c*$^{fl/fl}$ (P29)**	
	Ctrl	Cre+	Ctrl	Cre+
Resting potential (mV)	-74.4 ± 0.9 (6)	-72.8 ± 2.0 (7)	-67.0 ± 1.7 (6)*[2]	-72.8 ± 0.5 (5)*[2]
Membrane capacitance (pF)	10.6 ± 0.8 (5)*[1]	8.6 ± 0.3 (6)*[1]	10.2 ± 0.7 (6)*[3]	7.3 ± 0.2 (4)*[3]
I_{K1} at -124 mV (pA)	-	-271 ± 30 (6)	-	-128 ± 6 (4)
I_K at 0 mV (nA)	8.0 ± 0.7 (5)	8.0 ± 0.2 (6)	16.7 ± 1.0 (6)*[4]	9.2 ± 0.8 (4)*[4]
Adult type K$^+$ currents				
$I_{K,f}$ at -25 mV (nA)	1.3 ± 0.1 (5)	-	2.3 ± 0.2 (6)	-
$I_{K,n}$ at -124 mV (pA)	442 ± 33 (4)	-	327 ± 50 (6)	-

Values are means ± s.e.m.; number of hair cells are in parentheses. I_{K1} = Inward rectifier K$^+$ current[1]; I_K = Delayed rectifier K$^+$ current[2]; $I_{K,n}$ = Negatively activated K$^+$ current carried by KCNQ4 channels[2]; $I_{K,f}$ = Ca^{2+}-activated K$^+$ current[3]. "-" = not present. The size of the outward I_K (steady-state at 0 mV) and the inward I_{K1} (steady-state at –124 mV) was obtained from holding potentials of –84 mV and –64 mV, respectively. The size of $I_{K,f}$ (at –25 mV and at ~1 ms from the stimulus onset) and $I_{K,n}$ (difference between the peak and steady state of the deactivating inward current at -124 mV) was obtained from holding potentials of –84 mV and –64 mV, respectively. The above data were recorded from: two *Ush1c*$^{fl/fl}$ mice at both P14-P15 and P29; five P14-P15 and two P29 *Ush1c*$^{fl/fl}$*Myo15*-cre$^{+/-}$ mice. Note that in adult IHCs, the characteristic K$^+$ currents ($I_{K,f}$ and $I_{K,n}$) are absent in knockout mice (*Ush1c*$^{fl/fl}$*Myo15*-cre$^{+/-}$). Comparisons between *Ush1ca*$^{fl/fl}$ (Ctrl, left columns) and *Ush1c*$^{fl/fl}$*Myo15*-cre$^{+/-}$ (Cre+, right columns) were performed using the Student's *t* test:*[1]P=0.0286; *[2]P=0.0139; *[3]P=0.0148; *[4]P=0.0007.

Supplementary Table 3. Basolateral membrane properties from IHCs of *Myo7a*$^{fl/fl}$ conditional knockout mice

Adult

	***Myo7a*$^{fl/fl}$ (P14-P15)**		***Myo7a*$^{fl/fl}$ (P19-P22)**		***Myo7a*$^{fl/fl}$ (P50-59)**		***Myo7a*$^{fl/fl}$ (P210-218)**	
	Ctrl	Cre+	Ctrl	Cre+	Ctrl (P59)	Cre+ (P50-P59)	Ctrl (P210)	Cre+ (P218)
Resting potential (mV)	-73.4 ± 1.7 (8)	-72.1 ± 0.9 (8)	-71.0 ± 1.0 (13)	-72.0 ± 1.4 (12)	-71.2 ± 2.6 (4)	-67.0 ± 1.2 (7)	-73.6 ± 1.8 (5)	-69.9 ± 3.5 (4)
Membrane capacitance (pF)	9.3 ± 0.3 (9)	9.1 ± 0.3 (8)	10.1 ± 0.4 (14)	10.0 ± 0.4 (14)	9.9 ± 1.0 (4)*[3]	6.0 ± 0.4 (7)*[3]	6.4 ± 0.2 (5)*[5]	5.3 ± 0.4 (4)*[5]
I_{K1} at -124 mV (pA)	-	-	-	-	-	36 ± 7 (5)	-	31 ± 9 (3)
I_K at 0 mV (nA)	10.9 ± 1.3 (9)	11.0 ± 0.7 (8)	13.4 ± 1.1 (11)	13.3 ± 1.1 (13)	13.2 ± 0.7 (4)	11.9 ± 1.1 (7)	8.9 ± 0.9 (5)	7.4 ± 0.7 (4)
Adult type K$^+$ currents								
$I_{K,f}$ at -25 mV (nA)	0.9 ± 0.1 (9)	0.9 ± 0.1 (8)	1.9 ± 0.2 (11)*[1]	1.3 ± 0.1 (13)*[1]	1.8 ± 0.1 (4) *[4]	0.2 ± 0.1 (7)*[4]	1.4 ± 0.1 (5)*[6]	0.2 ± 0.1 (4)*[6]
$I_{K,n}$ at -124 mV (pA)	224 ± 18 (8)	166 ± 18 (8)	275 ± 21 (11)*[2]	200 ± 16 (11)*[2]	234 ± 63 (4)	-	219 ± 24 (5)	-

Values are means ± s.e.m.; number of hair cells are in parentheses. I_{K1} = Inward rectifier K$^+$ current[1]; I_K = Delayed rectifier K$^+$ current[2]; $I_{K,n}$ = Negatively activated K$^+$ current carried by KCNQ4 channels[2]; $I_{K,f}$ = Ca^{2+}-activated K$^+$ current[3]. "-" = not present. The size of the outward I_K

(steady-state at 0 mV) and the inward I_{K1} (steady-state at –124 mV) was obtained from holding potentials of –84 mV and –64 mV, respectively. The size of $I_{K,f}$ (at –25 mV and at ~1 ms from the stimulus onset) and $I_{K,n}$ (difference between the peak and steady state of the deactivating inward current at -124 mV) was obtained from holding potentials of –84 mV and –64 mV, respectively. The above data were recorded from: 4 (P14-P15), 7 (P19-P22), 5 (P29-P30), 2 (P59) and 1 (P210) *Myo7a*$^{fl/fl}$ mice; 4 (P14-P15), 8 (P19-P22), 2 (P29-P30), 3 (P59) and 1 (P218) *Myo7a*$^{fl/fl}$*Myo15*-cre$^{+/-}$ mice. Note that in adult mice, the characteristic K^+ currents ($I_{K,f}$ and $I_{K,n}$) are absent in P50-59 and P210-P218 IHCs from knockout mice (*Myo7a*$^{fl/fl}$*Myo15*-cre$^{+/-}$), which instead express an immature current profile. Comparisons between *Myo7a*$^{fl/fl}$ (Ctrl, left columns) and *Myo7a*$^{fl/fl}$*Myo15*-cre$^{+/-}$ (Cre+, right columns) were performed using the Student's *t* test: *[1]P=0.045; *[2]P=0.002; *[3]P=0.0164; *[4]P<0.0001; *[5]P<0.042; *[6]P<0.0001.

Supplementary Table 4. Basolateral membrane properties from *Myo15-cre*$^{+/-}$ mice

	***Myo15-cre*$^{+/-}$ (P99)**
Resting potential (mV)	-73.4 ± 0.8 (5)
Membrane capacitance (pF)	13.1 ± 1.0 (5)
I_{K1} at -124 mV (pA)	-
I_K at 0 mV (nA)	13.4 ± 0.8 (5)
Adult type K$^+$ currents	
$I_{K,f}$ at -25 mV (nA)	3.2 ± 0.1 (5)
$I_{K,n}$ at -124 mV (pA)	236 ± 25 (5)

Values are means ± s.e.m.; number of hair cells are in parentheses. I_{K1} = Inward rectifier K^+ current[1]; I_K = Delayed rectifier K^+ current[2]; $I_{K,n}$ = Negatively activated K^+ current carried by KCNQ4 channels[2]; $I_{K,f}$ = Ca^{2+}-activated K^+ current[3]. "-" = not present.

Supplementary Figure 1

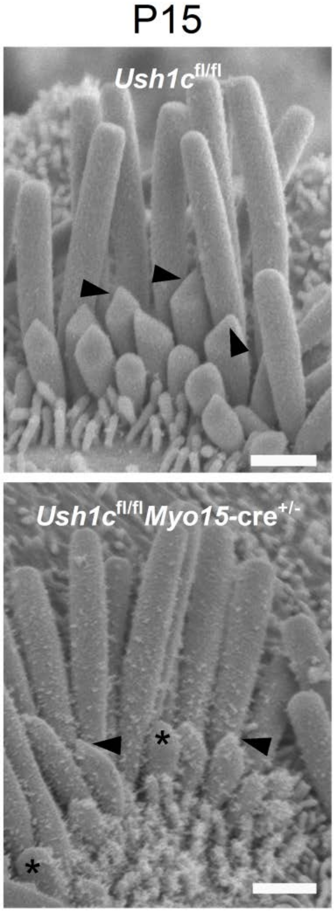

Supplementary Figure 1. Scanning electron microscopy (SEM) images from IHC stereocilia of P15 *Ush1c*$^{fl/fl}$ conditional knockout mice

SEM images showing the stereociliary bundles of apical-coil IHCs from P15 control *Ush1c*$^{fl/fl}$ (top) and *Ush1c*$^{fl/fl}$*Myo15*-cre$^{+/-}$ (bottom) mice. Scale bars, 1 µm. Note that prolate-shaped tips are present in all second row stereocilia of the control hair bundle (top, arrowheads point to some of them) but not in the *Ush1c*$^{fl/fl}$*Myo15*-cre$^{+/-}$ (bottom); some stereocilia have a more rounded tip (asterisks), which is similar to the finding in P25 IHCs using TEM (**Fig. 2f**).

Supplementary Figure 2

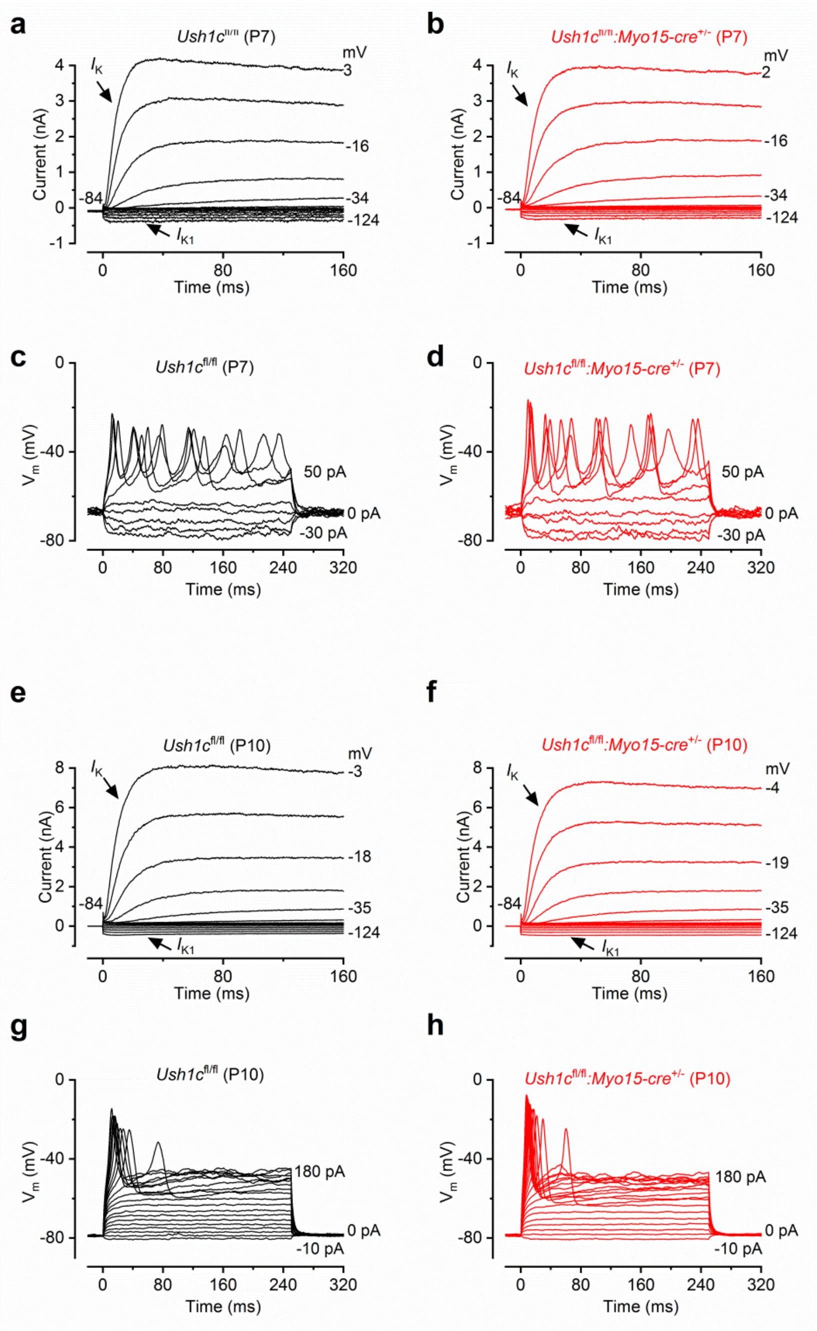

Supplementary Figure 2. Basolateral membrane properties from IHCs of pre-hearing *Ush1c*$^{fl/fl}$ conditional knockout mice

(**a,b**) Potassium currents recorded from P7 immature IHCs of control *Ush1c*$^{fl/fl}$ (**a**) and littermate *Ush1c*$^{fl/fl}$*Myo15*-cre$^{+/-}$ (**b**) mice using depolarizing voltage steps in 10mV nominal increments from the holding potential of –84 mV to the various test potentials shown by some of the traces. Note that IHCs from both genotypes show the same K^+ currents, which are characteristic of immature cells (I_K and I_{K1}). (**c,d**) Voltage responses elicited by applying

10 pA hyperpolarizing and depolarizing current injections to control *Ush1c*$^{fl/fl}$ (**c**) and *Ush1c*$^{fl/fl}$*Myo15*-cre$^{+/-}$ (**d**) immature IHCs from their respective membrane potentials. IHCs from both control *Ush1c*$^{fl/fl}$ and *Ush1c*$^{fl/fl}$*Myo15*-cre$^{+/-}$ IHCs exhibit Ca^{2+}-dependent action potentials following depolarizing current injections. (**e,f**) Potassium currents recorded from P10 immature IHCs of control *Ush1c*$^{fl/fl}$ (**e**) and littermate *Ush1c*$^{fl/fl}$*Myo15*-cre$^{+/-}$ (**f**) mice using the same protocol as in panels (**a**) and (**b**). Note a similar current profile in both genotypes. (**g,h**) Voltage responses from P10 immature IHCs of control *Ush1c*$^{fl/fl}$ (**g**) and *Ush1c*$^{fl/fl}$*Myo15*-cre$^{+/-}$ (**h**). Protocol as in panels **c** and **d**. For more details see **Supplementary Table 1.**

Supplementary Figure 3

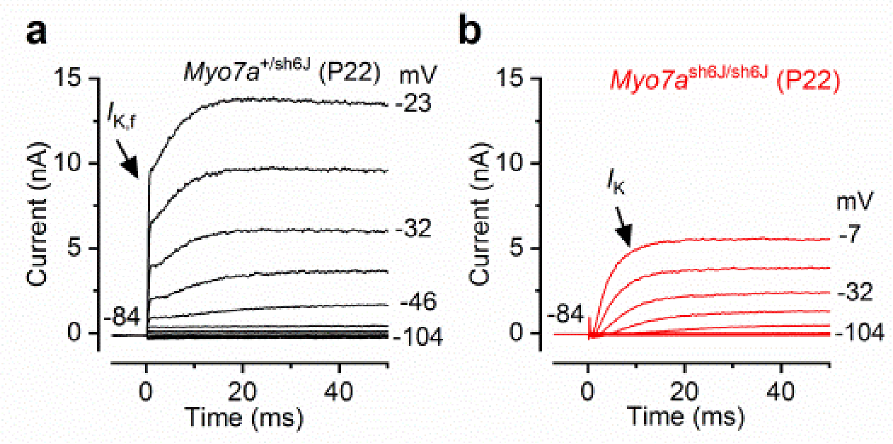

Supplementary Figure 3. IHCs from *Myo7a* mutant mice fail to become functionally mature

(**a,b**) Potassium currents recorded from adult (P22) IHCs from control and littermate *Myo7a* mutant mice (*Myo7a*$^{sh6J/sh6J}$)[4] using depolarizing voltage steps in 10 mV nominal increments from the holding potential of –84 mV to the various test potentials shown by some of the traces. Note that as for *Pcdh15* mutant IHCs (**Fig. 1**), the K^+ currents characteristic of adult IHCs ($I_{K,f}$ and $I_{K,n}$) are not present in *Myo7a*$^{sh6J/sh6J}$ cells, indicating that in the absence of these proteins IHCs are unable to become mature sensory receptors.

Supplementary Figure 4

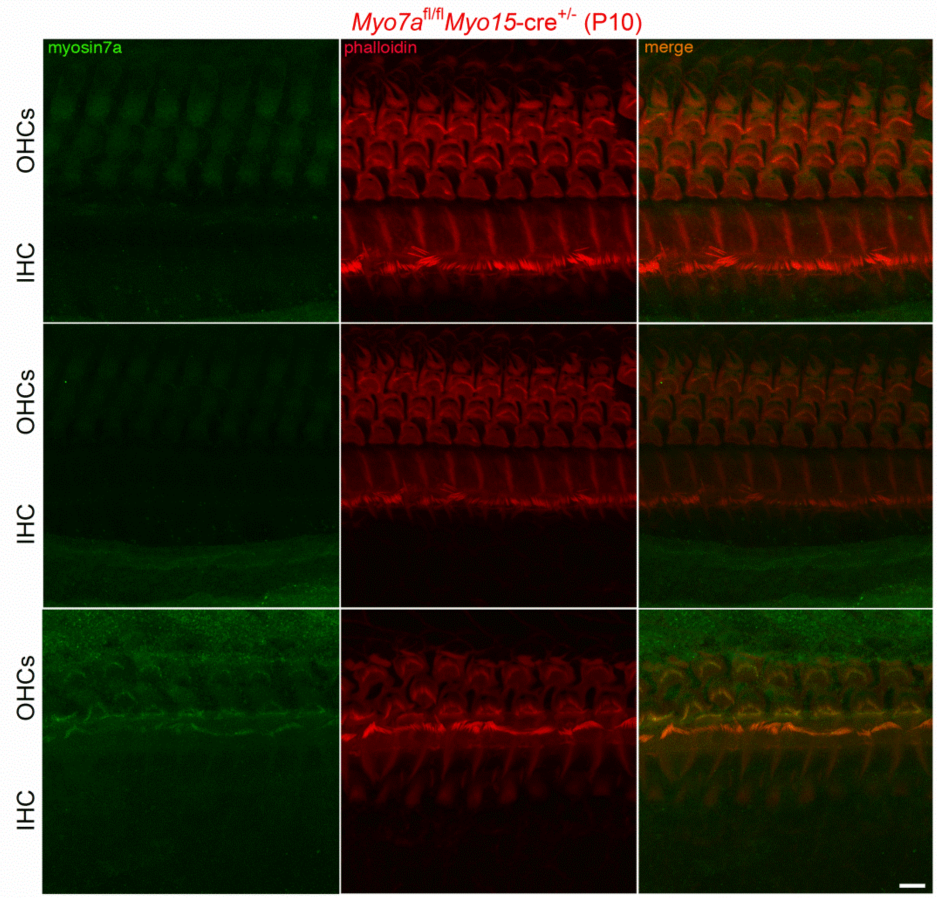

Supplementary Figure 4. Expression profile of Myo7a in IHCs from *Myo7a*$^{fl/fl}$ conditional knockout mice.

Additional examples of cochlear whole mount preparations from P10 *Myo7a*$^{fl/fl}$*Myo15-cre*$^{+/-}$ conditional knockout mice immunostained for Myo7a (green) and for F-actin with phalloidin (red). Note that Myo7a was detected in both OHCs and IHCs from one *Myo7a*$^{fl/fl}$*Myo15*-cre$^{+/-}$ mouse (lower panel). Scale bar = 5 µm.

Supplementary Figure 5

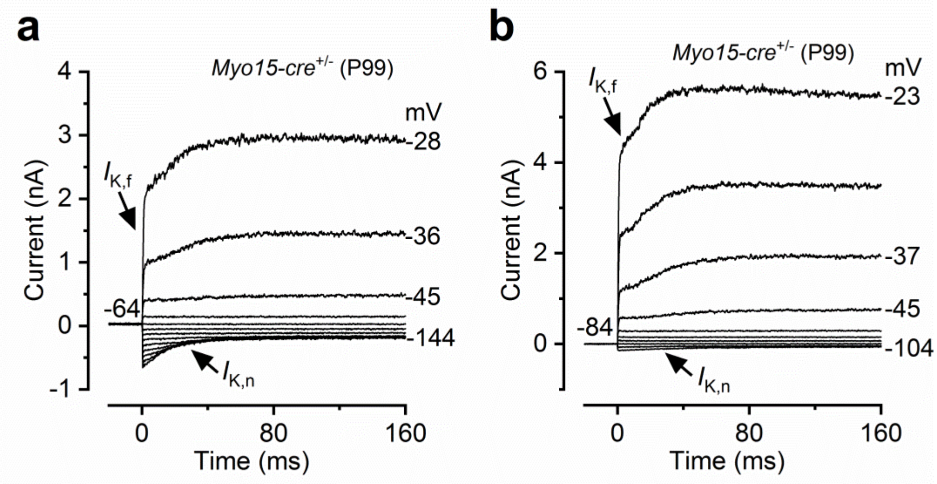

Supplementary Figure 5. IHCs from adult *Myo15-cre*$^{+/-}$ mice are normal

(**a,b**) Potassium currents recorded from an adult (P99) IHC of a *Myo15-cre*$^{+/-}$ mouse depolarizing voltage steps in 10 mV nominal increments from a holding potential of either −64 mV (**a**) or −84 mV (**b**) to the various test potentials shown by some of the traces. The two holding potentials were used in order to better compare the data from *Myo15-cre*$^{+/-}$ mice with those shown from *Myo7a*$^{fl/fl}$*Myo15-cre*$^{+/-}$ (−64 mV**: Figs. 4 and 5**; −84 mV**: Fig. 7**). Note the presence of the K^+ currents characteristic of adult IHCs ($I_{K,f}$ and $I_{K,n}$). Basolateral membrane properties for *Myo15-cre*$^{+/-}$ IHCs are listed in the **Supplementary Table 4.**

Supplementary Figure 6

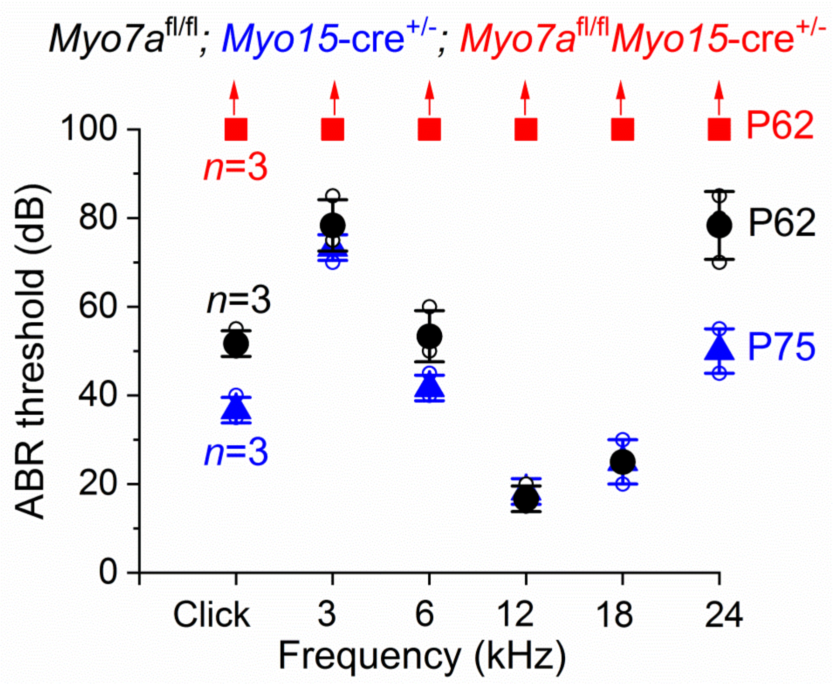

Supplementary Figure 6. ABRs from adult *Myo7a*$^{fl/fl}$ conditional knockout mice

Mean ABR thresholds (± s.d.) for clicks and frequency-specific pure tone stimulation from 3 kHz to 24 kHz obtained from control P62 *Myo7a*$^{fl/fl}$ and littermate *Myo7a*$^{fl/fl}$*Myo15*-cre$^{+/-}$ mice. Note that *Myo7a*$^{fl/fl}$*Myo15*-cre$^{+/-}$ mice deaf (arrows indicates no response at the maximum sound pressure level used). Mean ABR thresholds of P75 *Myo15*-cre$^{+/-}$ mice were comparable to those in *Myo7a*$^{fl/fl}$ mice.

Supplementary Figure 7

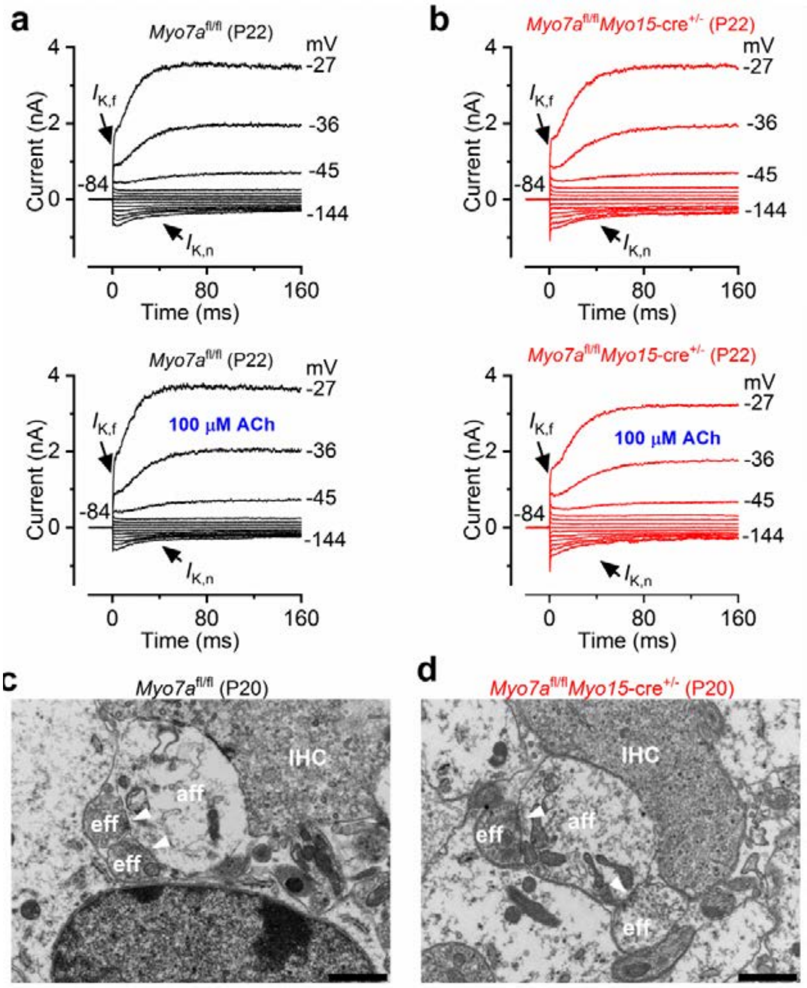

Supplementary Figure 7. Lack of responses to the efferent neurotransmitter ACh on IHCs from young adult *Myo7a*$^{fl/fl}$ conditional knockout mice

(**a,b**) Membrane currents recorded from adult IHCs of control *Myo7a*$^{fl/fl}$ (**a**) and *Myo7a*$^{fl/fl}$*Myo15*-cre$^{+/-}$ (**b**) mice before (top panels) and during superfusion of ACh (lower panels). Note that ACh does not activate any additional current at this young adult stage (P22). These are representative recordings from 5 P22 *Myo7a*$^{fl/fl}$ IHCs (2 mice) and 12 P22 *Myo7a*$^{fl/fl}$*Myo15*-cre$^{+/-}$ (3 mice). (**c,d**) Transmission electron microscopy showing the characteristic synaptic organization of mature P20 IHCs in both control *Myo7a*$^{fl/fl}$ (**c**) and *Myo7a*$^{fl/fl}$*Myo15*-cre$^{+/-}$ (**d**) mice; efferent terminals form axo-dendritic contacts with the afferent fibres, as seen by presence of active zones with synaptic vesicles (white arrows). Scale bars, 1 µm. Note that in *Myo7a*$^{fl/fl}$*Myo15*-cre$^{+/-}$ (**d**), one of the two efferent fibres gets closer to the IHC but without making synaptic contacts (no active zones or vesicles were present throughout the serial sections), which was also seen in a few control IHCs.

Supplementary references

1. Marcotti, W., Géléoc, G.S.G., Lennan, G.W.T. & Kros, C.J. Developmental expression of an inwardly rectifying potassium conductance in inner and outer hair cells along the mouse cochlea. *Pflugers Arch.* **439**, 113-122 (1999).
2. Marcotti, W., Johnson, S.L., Holley, M.C. & Kros, C.J. Developmental changes in the expression of potassium currents of embryonic, neonatal and mature mouse inner hair cells. *J. Physiol.* **548**, 383-400. (2003).
3. Kros, C.J., Ruppersberg, J.P. & Rüsch, A. Expression of a potassium current in inner hair cells during development of hearing in mice. *Nature* **394**, 281-284 (1998).
4. Kros, C.J. *et al.* Reduced climbing and increased slipping adaptation in cochlear hair cells of mice with Myo7a mutations. *Nat. Neurosci.* **5**, 41-47 (2002).
